# Safety Evaluation of Unani Formulation: Capsule Shaqeeqa in Albino Wistar Rats

**DOI:** 10.1155/2016/2683403

**Published:** 2016-03-31

**Authors:** Khalid Ghazanfar, Showkat Ahmad Dar, Seema Akbar, Tazeen Nazir, Mariya Hamdani, Khalid M. Siddiqui, Pawan Kumar, Akbar Masood

**Affiliations:** ^1^Drug Standardisation Research Unit, Regional Research Institute of Unani Medicine, CCRUM, University of Kashmir, Naseem Bagh Campus, Srinagar 190006, India; ^2^Department of Biochemistry, University of Kashmir, Srinagar, Jammu and Kashmir 190006, India; ^3^Central Council for Research in Unani Medicine, New Delhi 110058, India

## Abstract

Capsule Shaqeeqa, Unani formulation, is prescribed for the clinical treatment of diseases like sinusitis, headache, and migraine. The safety evaluation data of it is not available; in order to provide the safety data the present study was carried out. The study was carried out on four groups of rats (*n* = 5). Two groups (one male and one female group) as normal controls were orally given water while the other two groups were orally given daily doses of drug at the dose level of 150 mg/kg of body weight for duration of 90 days. Physiological parameters like body weight, feed consumption, water consumption, and clinical signs were regularly monitored and recorded. Organs were collected, examined, and weighed and specimens were taken for histopathological studies. The results showed that the drug did not alter the physiological parameters. There was no mortality or any morbidity found in drug treated rats. There was no statistical significant change found in any haematological or biochemical parameter of rats orally fed with Shaqeeqa. A statistically insignificant association verified that haematological and biochemical parameters were rendered unaffected by the drug. Moreover histological investigations of essential key organs demonstrated that the drug did not prompt any histopathological change. These observations demonstrate the safety of Capsule Shaqeeqa at the studied dosage levels.

## 1. Introduction

There are several traditional systems of medicines such as the Ayurveda, Unani, and Siddha which have been in use since times immemorial and have been a vast source for development of majority of modern medicines. The majority of ingredients found in these traditional systems of medicines are herbal in nature which has been reported to possess numerous pharmacological activities [1–3]. Modern drug development research relies on ethnobotany and ethnopharmacognosy for discovery of new molecules [4]. However, there is a widespread misconception regarding the use herbal medicines; that is, they are devoid of toxic effects [5]. Studies have proven that the herbal formulation is also associated with several side effects including allergic reactions, hepatotoxicity [6], nephrotoxicity [7–10], cardiac toxicity [11, 12], neurotoxicity [13, 14], and even death [15]. Hence a safety evaluation study is indispensable to validating traditional medicine safe medicinal use [16]. Safety evaluation studies endow necessary experimental data and information with respect to the scientific data and safety measures of a drug, thereby assuring their prescription by practitioners [5, 17, 18].

Capsule Shaqeeqa is Unani formulation used for treating headache, sinusitis, and migraine. The drug is composed of Ustukhuddus (*Lavandula stoechas* Linn.), Kishhiz khushk (*Coriandrum sativum* Linn.), and Filfil Siyah (*Piper nigrum* Linn.). The dosage of the drug is 500 mg (one capsule/day) [19]. The dose 150 mg/kg was selected as it corresponds to 3*X* of the rat extrapolated dose while *X* is the extrapolated dose for rats which itself corresponds to the seven times of human dose. The safety studies of all medicinal entities are very important and that is true for Unani formulations, as they not only ensure the acceptability of these preparations in a wider international community of medical practitioners and patients, but also would result in their wider usage. Since data regarding the toxicological evaluation of Capsule Shaqeeqa is not present in the literature, the present study assesses conceivable adverse effects of Capsule Shaqeeqa following Organization of Economic Cooperation and Development (OECD) guidelines.

## 2. Methods and Materials

### 2.1. Test Item

The drug Capsule Shaqeeqa was supplied by CRI, Hyderabad (CCRUM), with manufacturing date of October 2012. The drug was stored in a moisture-free place and a sample of the drug was archived for records. The suspension of the drug was prepared using water.

### 2.2. Animals and Maintenance

Adult albino Wistar rats of both sexes were used in the study. Animals were housed in polypropylene cages with stainless steel grid covers and wood shavings as bedding material, in an ambient environment. The rats were provided with pelleted feed and Reverse Osmosis (RO) water* ad libitum*. Research on animals was conducted in accordance with the guidelines of the Committee for the Purpose of Control and Supervision of Experiments on Animals (CPCSEA) as the institute has CPCSEA registration (Reg. number 927/GO/c/06/CPCSEA). The experimental protocol was approved by the Institutional Animal Ethics Committee of the Regional Research Institute of Unani Medicine, Srinagar, Jammu and Kashmir, India.

### 2.3. Experimental Design

The subchronic oral toxicity was conducted in accordance with the OECD guideline [20]. The rats were randomly divided into 4 groups; each group consisted of 5 rats. Groups I and II being the male and female controls were orally given water (vehicle). Groups III and IV being the drug treated male and female rats were orally given Capsule Shaqeeqa at the dose of 150 mg/kg body weight for 90 days daily. All the animals were closely examined for any adverse toxic signs, behavioural changes, and so forth. The body weight of the rats was recorded weekly. Food and water consumption/rat/24 hours were recorded weekly. On the 91st day, after overnight fasting, all the animals were sacrificed by exsanguination by withdrawing blood in a syringe from the dorsal vena cava after opening the abdomen under ISOFLURANE anaesthesia. Two millilitres of blood was added to EDTA vacutainer for the study of haematological parameters and 3 mL blood was added to red tap vacutainer containing the clotting activators. The clotted blood was centrifuged and the serum was separated for the study of biochemistry parameters. All the animals were dissected to check macroscopic morphology of the body organs/tissues. The organs such as liver, lung, kidney, adrenal gland, pancreas, spleen, brain, ovary/testes, and heart were collected to determine the organ weight followed by grossing of tissues for histopathological studies.

### 2.4. Biochemistry Parameters

Biochemical parameters were studied in serum obtained after centrifugation of blood at 2000 RPM for 15 minutes on the day of the rat sacrifice. Biochemical parameters were determined on fully automatic biochemistry analyzer (XL640 TRANSASIA) using ERBA kits. Liver function tests, aspartate aminotransferase (AST), alanine aminotransferase (ALT), alkaline phosphotase (ALP), total bilirubin, total protein, and albumin, and kidney function tests, blood urea, uric acid, and other biochemical substances such glucose, cholesterol, and triglycerides were estimated.

### 2.5. Haematological Parameters

Haematological parameters were analyzed in freshly collected blood in blue top vacutainer containing EDTA anticoagulant. The blood was gently mixed with the EDTA anticoagulant coated on the tube walls. Haematological parameters were determined on fully automatic haematological analyzer (Sysmex XT2000iV, Sysmex Corporation, Japan). Haematological parameters such as haemoglobin conc., WBC count, RBC count, haematocrit value, mean corpuscular volume, mean corpuscular haemoglobin concentration, mean corpuscular haemoglobin, platelet count, differential leukocyte count, neutrophil%, lymphocyte%, monocyte%, eosinophil%, and basophil%, and reticulocyte count were studied.

### 2.6. Histopathology

Tissue samples were collected from the organs of control as well as treated male/female rats of the subchronic study. The tissues collected from the organs such as liver, lung, kidney, pancreas, spleen, brain, ovary/testes, and heart were numbered for identification and then transferred to tissue cassettes (SS) to enable fixation in 10% formalin for 36–48 hours followed by the tissue processing which was carried out on automatic tissue processor Model No1020 (LEICA make, Germany). The tissue processing included dehydration in graded isopropyl alcohol, clearing in xylene I and xylene II, impregnation in paraffin wax, and finally tissue blocks which were prepared on paraffin block maker (Model number 1150H+C, LEICA make, Germany). Section cutting of tissue blocks was done using microtome (YORCO) to the thickness of 5–8 microns. The tissue sections were fixed on the slide by heat technique followed by the staining by haematoxylin and eosin stain. The staining was carried on automatic slide stainer (THERMO MAKE, Germany) using haematoxylin and eosin staining. After staining the tissue sections were mounted with DPX to prevent any damage to the stained tissue. The stained tissue sections were examined under microscope 40x objective to check the adverse effects of drug on cell morphology as well as on the cell organelles.

### 2.7. Statistical Analysis

All the values of body weight, biochemical estimations, haematological parameters, feed intake values, and water intake values were expressed as *X*  ±  SEM and analyzed for one-way ANOVA using SPSS 16.0 statistical software. Differences between groups were considered significant at *p* < 0.05 levels.

## 3. Results

### 3.1. Effect of Drug on Feed and Water Consumption of Rats

The average feed consumption in control males was found to be 16.6 g while the drug treated male group was recorded as 16.9 g (Table 1). The mean values of all the three groups are quite comparable as there are minor differences. Similarly the mean feed consumption in control female group was found as 16.1 g and drug treated female group was found as 16.5 g.

The average water consumption of control males was found as 26.1 mL, while the drug treated male group was found to be 26.6 mL. The average water consumption of drug treated female group was found to be in the close range of control female group as shown in Table 1.

### 3.2. Effect of Drug on the Body Weight of Rats

The mean body weight gain (%) of the entire drug treated male and female groups was found to be not so different from the respective male and female controls. The rat groups treated with drug were found to grow and gain body weight in a normal fashion. The average body weight gain of these treated rat groups was found to be unaffected by the extracts as depicted in Table 2.

### 3.3. Effect of Drug on the Organ/Tissue Weights of Rats

There was no statistically significant difference in the weight of tissue/organs of drug treated rats and control rats (Table 3). There was no morphological change observed in tissues/organs of treated rats at necropsy. The collected tissues/organs were found to be normal in appearance, size, shape, and texture when compared with the normal controls.

### 3.4. Effect of Drug on Various Biochemical Parameters of Rats

The results of biochemical parameters did not show any significant change in the levels when compared with the controls. The liver and kidney function test parameters were found to be normal in the drug treated groups. The lipid profiles of the drug treated male and female rats were found to be unaffected by the drug administration when compared with respective controls as shown in Table 4.

### 3.5. Effect of the Drug on Various Haematological Parameters of Rats

The drug was found to have no significant change in the haematological parameters such as WBC counts, total leukocyte count, haemoglobin, haematocrit, total erythrocyte count, erythrocyte indices (MCV, MCH, and MCHC), and platelets count of both male and female groups as shown in Table 5.

### 3.6. Effect of the Drug on Histopathology of Various Organs/Tissue

**Figure 1:**
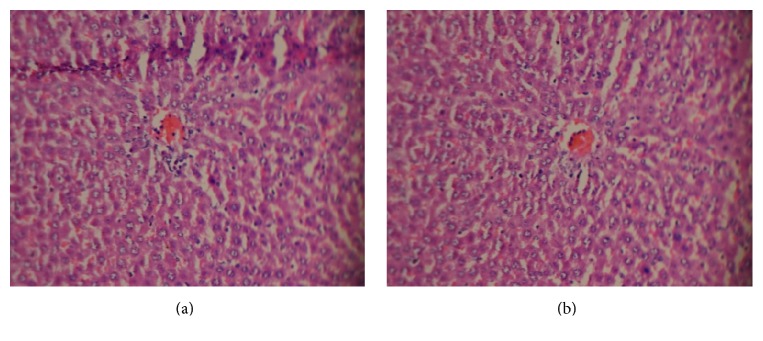
(a) Treated liver. (b) Control liver.

**Figure 2 fig2:**
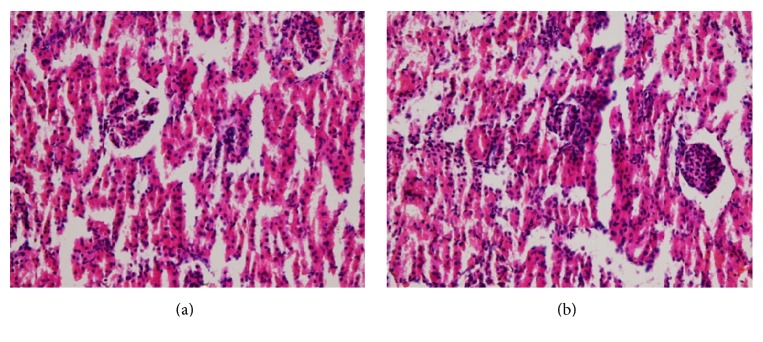
(a) Treated kidney. (b) Control kidney.

**Figure 3 fig3:**
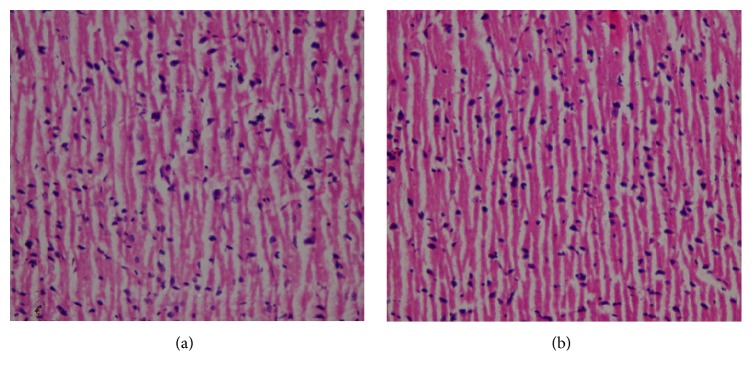
(a) Treated brain. (b) Control brain.

**Figure 4 fig4:**
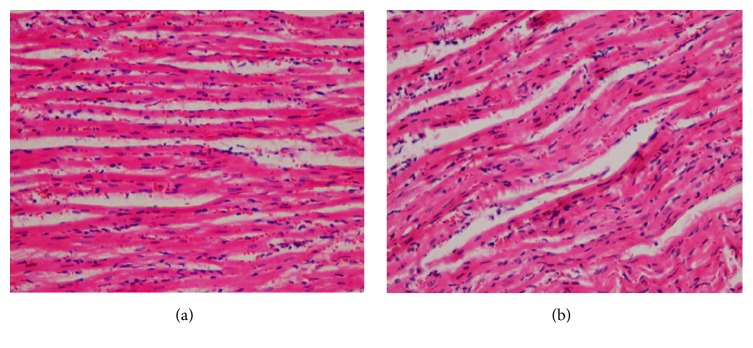
(a) Treated heart. (b) Control heart.

**Figure 5:**
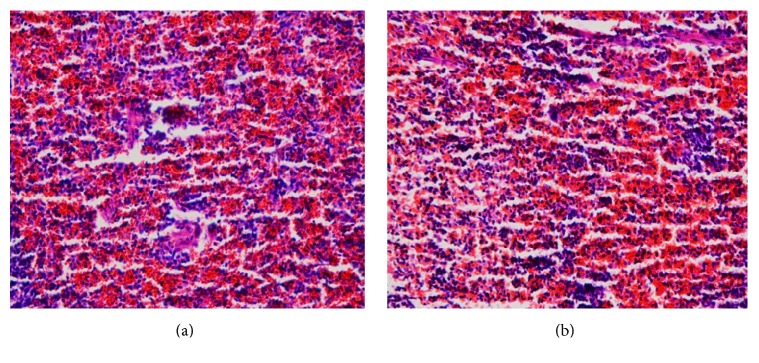
(a) Treated spleen. (b) Control spleen.

There were no treatment related pathological changes found in the brain, heart, lung, liver, kidney, spleen, adrenal, testes, and ovaries of the rats at the dose level tested. No pathological change was found in the collected organs/tissues of drug treated rats as shown in figures. Microscopic examination of all the organs obtained from control group exhibited normal cytoarchitecture. Figures 1 [Fig fig2] [Fig fig3] [Fig fig4]–5 show different slides of drug treated and control tissues; as observable there is no major difference in treated and control slides; the photomicrographs were taken at 40x.

## 4. Discussion

There is well-established literature regarding the efficacy of traditional medicinal formulations; however the safety data of them is yet to be established. Traditional medicinal formulations including herbal products at times have been proved to be toxic with severe adverse effects [21, 22]. It is nowadays fundamental to establish the safety data of drugs/formulations in order to ensure their safe use. The methods of the development of traditional medicines need to be validated on scientific guidelines, and that is true for establishment of safety of these medicines [23, 24].

Keeping in view the importance of safety evaluation of traditional system of medicines, the current study was carried out to establish the safety data in rats. The drug Capsule Shaqeeqa was proven to be nonlethal as neither report of mortality nor any morbidity was recorded at the dose level selected. One of the major signs of toxicity is reduction in body growth and weight [25]; a normal pattern of growth and body weight gain was found in drug treated rats when compared with the controls. There was no change observed in the gross behaviour of the rats orally fed with the drug for 90 days. A statistically nonsignificant change in the biochemical parameters of the treated male and female rats was found. There was no abnormality found in liver marker enzymes. The kidney function tests were also found to be normal in drug treated rats. One of the most sensitive targets for toxic entities is haematopoietic system; thus it is very much mandatory to observe any possible abnormalities resulting from a test substance [26, 27]. There was a nonsignificant change observed in the haematological parameters of drug treated males and females when compared with the respective controls. The histopathological study of Capsule Shaqeeqa treated male and female showed that the drug is safe as there was no abnormality in the cytoarchitecture of the tissues/organs collected.

## 5. Conclusion

Capsule Shaqeeqa was found to be safe in subchronic toxicity studies at the selected dose level in male and female rats when given repeatedly for 90 days orally, as there were no statistically significant changes found in behaviour, physiological, biochemical, and haematological parameters. There was no adverse effect of the drug found in rats.

## Figures and Tables

**Table 1:** Average feed and water consumption of rats.

Groups	Average feed consumption (g/day)		Average water consumption (ml/day)	
	Control	Treated	Control	Treated
Male	16.6 ± 0.29	16.9 ± 0.27	26.1 ± 0.34	26.9 ± 0.47
Female	16.1 ± 0.44	16.5 ± 0.39	25.8 ± 0.55	26.1 ± 0.42

The values are expressed as mean ± SEM. *n* = 5 in each group.

*p* < 0.05 as compared with controls (one-way ANOVA).

**Table 2:** Effect of Shaqeeqa on body weight of female rats.

Parameter	Control male	Treated male	Control female	Treated female
% gain in body weight	74.59	75.19	68.39	63.28

The values are expressed as mean ± SEM. *n* = 5 in each group.

*p* < 0.05 as compared with controls (one-way ANOVA).

**Table 3:** Relative organ/tissue weights of male and female rats.

Organ/tissue	Control male	Treated male	Control female	Treated female
Brain (g)	0.84 ± 0.04	0.82 ± 0.04	0.76 ± 0.02	0.77 ± 0.03
Lungs (g)	0.79 ± 0.04	0.77 ± 0.03	0.68 ± 0.03	0.65 ± 0.04
Heart (g)	0.34 ± 0.02	0.35 ± 0.03	0.29 ± 0.01	0.31 ± 0.02
Liver (g)	3.88 ± 0.18	3.68 ± 0.13	3.39 ± 0.11	3.54 ± 0.18
Spleen (g)	0.42 ± 0.03	0.43 ± 0.03	0.36 ± 0.02	0.35 ± 0.02
Pancreas (g)	0.29 ± 0.02	0.25 ± 0.02	0.23 ± 0.01	0.24 ± 0.02
Kidney (g)	0.39 ± 0.03	0.36 ± 0.04	0.33 ± 0.01	0.34 ± 0.03
Adrenal (g)	0.01 ± 0.0	0.01 ± 0.0	0.01 ± 0.0	0.01 ± 0.0
Testis (g)/ovaries (g)	0.52 ± 0.02	0.55 ± 0.02	0.02 ± 0.01	0.02 ± 0.00

The values are expressed as mean ± SEM. *n* = 5 in each group.

*p* < 0.05 as compared with controls (one-way ANOVA).

**Table 4:** Biochemical parameters of male and female rats.

Parameters	Control male	Treated male	Control female	Treated female
SGPT (IU/L)	78.4 ± 5.6	69.50 ± 7.19	80.8 ± 7.3	78.97 ± 6.7
SGOT (IU/L)	154.04 ± 8.75	138.73 ± 9.44	141 ± 7.6	137.37 ± 8.34
ALP (IU/L)	151 ± 6.36	144.75 ± 5.41	153.3 ± 8.57	164.52 ± 9.19
BIT (mg/dl)	0.09 ± 0.01	0.11 ± 0.01	0.12 ± 0.03	0.11 ± 0.02
UREA (mg/dl)	55.3 ± 1.85	61.10 ± 2.24	53.8 ± 2.2	50.37 ± 2.12
CRE (mg/dl)	0.81 ± 0.01	0.78 ± 0.01	0.72 ± 0.02	0.69.57 ± 0.1
UA (mg/dl)	2.55 ± 0.1	2.11 ± 0.12	2.58 ± 0.16	2.36 ± 0.21
TRIG (mg/dl)	70.2 ± 4.87	67.75 ± 5.94	83.1 ± 5.34	74.54 ± 4.31
CHO (mg/dl)	57.6 ± 5.67	61.25 ± 3.69	63.8 ± 3.8	55.19 ± 5.53
GLU (mg/dl)	83.3 ± 5.99	73.70 ± 4.57	79.4 ± 5.6	77.38 ± 5.78
ALB (g/dl)	4.2 ± 0.14	4.08 ± 0.21	4.56 ± 0.11	4.34 ± 0.17
PRO (g/dl)	7.51 ± 0.22	7.76 ± 0.33	7.26 ± 0.12	7.13 ± 0.24
HDLC (mg/dl)	31.92 ± 3.22	33.53 ± 3.17	34.73 ± 3.57	35.76 ± 3.94

The values are expressed as mean ± SEM. *n* = 5 in each group.

*p* < 0.05 as compared with controls (one-way ANOVA).

**Table 5:** Haematological parameters of male and female rats.

Parameters	Control male	Treated male	Control female	Treated female
WBC (10^3^/*µ*l)	10.24 ± 0.92	10.55 ± 0.86	11.59 ± 0.73	10.11 ± 0.93
RBC (10^6^/*µ*l)	8.66 ± 0.33	8.46 ± 0.29	8.23 ± 0.22	9.12 ± 0.23
HGB (g/dl)	14.62 ± 0.82	15.50 ± 0.60	15.73 ± 0.62	15.17 ± 0.78
HCT (%)	44.7 ± 1.54	45.55 ± 1.75	46.53 ± 1.57	45.03 ± 0.78
MCV (fl)	56.2 ± 0.87	53.90 ± 0.62	56.7 ± 0.24	59.13 ± 1.13
MCH (pg)	18.81 ± 0.25	18.35 ± 0.15	17.93 ± 0.48	18.43 ± 0.29
MCHC (g/dl)	30.8 ± 0.28	34.00 ± 0.18	31.7 ± 0.42	31.20 ± 0.48
PLT (10^3^/*µ*l)	1202 ± 42.46	1320 ± 50.37	1333.33 ± 48.73	1239 ± 67.10
NEUT (%)	9.3 ± 1.22	7.6 ± 1.51	8.36 ± 1.10	9.07 ± 1.96
LYMP (%)	80.5 ± 3.03	74.2 ± 5.83	78.9 ± 1.15	77.70 ± 1.04
MONO (%)	6.27 ± 0.46	6.4 ± 0.51	7.36 ± 0.68	7.20 ± 0.53
EO (%)	1.76 ± 0.1	1.7 ± 0.15	1.13 ± 0.13	1.77 ± 0.12
BASO (%)	0.4 ± 0.01	0.40 ± 0.05	0.43 ± 0.02	0.37 ± 0.05
RET (%)	3.13 ± 0.35	3.20 ± 0.25	3.52 ± 0.27	3.10 ± 0.18

The values are expressed as mean ± SEM. *n* = 5 in each group.

*p* < 0.05 as compared with controls (one-way ANOVA).
